# Predictive Value of Single Nucleotide Polymorphisms of *ERCC1*, *XPA*, *XPC*, *XPD* and *XPG* Genes, Involved in NER Mechanism in Patients with Advanced NSCLC Treated with Cisplatin and Gemcitabine

**DOI:** 10.1007/s12253-018-0459-8

**Published:** 2018-07-31

**Authors:** Radosław Mlak, Paweł Krawczyk, Iwona Homa-Mlak, Tomasz Powrózek, Marzanna Ciesielka, Piotr Kozioł, Janusz Milanowski, Teresa Małecka-Massalska

**Affiliations:** 1 Department of Human Physiology, Medical University of Lublin, Radziwiłłowska 11, 20-080 Lublin, Poland; 2 Department of Pneumology, Oncology and Allergology, Medical University of Lublin, Jaczewskiego 8, 20-954 Lublin, Poland; 3 Department of Forensic Medicine, Medical University of Lublin, Jaczewskiego 8b, 20-090 Lublin, Poland

**Keywords:** Non-small cell lung cancer, Polymorphism, DNA repair, XPD, chemotherapy

## Abstract

The combination of cisplatin and gemcitabine is still one of the most frequently used first-line chemotherapy scheme in patients with advanced non-small cell lung cancer (NSCLC), in which tyrosine kinase inhibitors (TKIs) cannot be administered. Unfortunately, more than half of the patients have no benefit from chemotherapy but are still exposed to its toxic effects. Therefore, single nucleotide polymorphisms (SNPs) in the genes involved in nucleotide excision repair (NER) mechanism may be a potential predictive factor of efficiency of cytostatic based chemotherapy. The aim of the study was to evaluate the correlation between SNPs of the genes involved in NER mechanism and the effectiveness of chemotherapy based on cisplatin and gemcitabine in patients with advanced NSCLC. The study group included 91 NSCLC patients treated with first-line chemotherapy using cisplatin and gemcitabine. Genotyping was carried out using a mini-sequencing technique (SNaPshot™ PCR). The median progression-free survival (PFS) was significantly shorter in carriers of CC genotype of the *XPD/ERCC2* (2251A > C) gene compared to patients with AA/AC genotypes (2 vs. 4.5 months; *p* = 0.0444; HR = 3.19, 95%CI:1.03–9.91). Rare CC genotype of *XPD/ERCC2* gene, may be considered as an unfavorable predictive factor for chemotherapy based on cisplatin and gemcitabine in patients with advanced NSCLC.

## Introduction

Lung cancer is a leading cause of cancer-related deaths in the developed countries. In the world, more than 1.5 million new cases and deaths due to lung cancer are reported every year. Non-small cell lung cancer (NSCLC) is the most frequent histopathological type of lung cancer and it is diagnosed in about 85% patients. Usually NSCLC is diagnosed in locally advanced or advanced stage of progression (IIIA – inoperable cases, IIIB, IV), which disqualifies these patients from radical surgery. Standard chemotherapy, often combined with radiotherapy, remains the main method of treatment. Standard first-line chemotherapy in patients with advanced NSCLC is based on combination of platinum compounds (mainly cisplatin) with a second generation drug (e.g. gemcitabine). However, most cytostatic-based treatment regimens used in NSCLC are characterized with similarly poor effectiveness. Objective response rate (ORR) is obtained usually in less than 40% of cases, median of overall survival (OS) increases only up to 1.5 months in comparison with best supportive care. On the other hand, it is usually associated with high systemic toxicity (nephro-, hepato- and haematological toxicity) [1–3].

More benefit for lung cancer patients is provided by molecularly targeted therapy (e.g. erlotinib, afatinib, crizotinib). Despite the fact, that we already have several generations of targeted drugs, this type of treatment can be used only in selected patients with specific genetic abnormalities: activating mutations in *EGFR* (9–51% depends by race) or *ALK* rearrangements (3–7%). A new promising tool for lung cancer therapy is the immune checkpoint inhibition, especially focused on programmed death-1 (PD-1)/programmed death ligand −1 (PD-L1) signalization [4–6]. Nevertheless, despite undeniable breakthrough related with the introduction of new molecularly targeted drugs, a substantial number of patients still receive cytostatics as a part of multidisciplinary treatment. Due to this fact, this type of therapy is addressed predominantly to patients diagnosed with adenocarcinoma, in whom the above mentioned rare molecular disorders occur more frequently [4, 5]. Therefore, it appears to be an interesting alternative to investigate new therapeutic targets and agents to maximize the benefits from the “old”, well known, cytostatics.

Platinum compounds react with DNA to form intra- as well as inter-strand cross-links. Then, the formed abnormal DNA structure (the so-called DNA adducts) may cause DNA strand breaks, inhibition of transcription and alterations of proteins encoding, which, in most cases, lead to apoptosis. Regarding gemcitabine mechanism of action, it is based on its incorporation into nucleic acids, which subsequently leads to inhibition of DNA replication and may also induce apoptosis [7].

Over thirty specialized proteins (including: *ERCC1*, *XPA*, *XPC*, *XPD* and *XPG*) are involved at various stages of NER mechanism, which is one of the major mechanism of DNA repair systems. Therefore, any change regarding their expression level and functioning (e.g. single nucleotide polymorphisms - SNPs) may lead to alteration of effectiveness of cytostatics (if their mechanism of action is based on direct or indirect DNA damage) [8].

Studies recorded over the past few years demonstrate that, in some subgroups of NSCLC patients receiving standard chemotherapy, genetic predisposition (e.g. SNPs), especially in genes encoding DNA repair proteins, may have the potential to become predictive or prognostic factors [9–12]. However, we still do not have any conclusive results and also their role as predictive or prognostic factors has not been definitively clarified.

The aim of this study was the assessment of the relationship between 8 SNPs of 5 genes involved in NER mechanism (*ERCC1*, *XPA*, *XPC*, *XPD* and *XPG*) and the effectiveness of cisplatin and gemcitabine based chemotherapy in patients with advanced NSCLC.

## Materials and Methods

### Study Group

Our study group (*n* = 91) included 67% male and 33% female Caucasian patients with NSCLC, recruited in 2010–2013 at the Department of Pneumonology, Oncology and Allergology, Medical University of Lublin. Each patient’s detailed demographic and clinical data were collected. The stage of disease was determined based on the TNM classification (VII edition by UICC): 30.8 and 69.2% of patients were in stage IIIB and IV, respectively. The basic inclusion criterion was first-line chemotherapy treatment based on platinum compounds and gemcitabine (median cycles of chemotherapy was 4). Detailed data of patients are specified in Table 1. The study protocol was approved by the Committee of Ethics and Research at the Medical University of Lublin (approval no.: KE-0254/142/2010). The informed consent was obtained from all individual participants enrolled in the study.

**Table 1:** Characteristics of the studied group

Variable	Study group (*n* = 91)
Sex	
Male Female	61 (67%) 30 (33%)
Age (years)	
Median Mean ± std. dev. Range	62 62.5 ± 7.9 38–78
Smoking status (pack-years)	
Median Mean ± std. dev. Non-smokers Current smokers Former Smokers No data	30 31.4 ± 9.5 5 (5.5%) 65 (71.4%) 20 (22%) 1 (1.1%)
Histopathological diagnosis	
Adenocarcinoma Squamous cell carcinoma Large cell carcinoma NOS (not otherwise specified)	46 (50.5%) 14 (15.4%) 16 (17.6%) 15 (16.5%)
Stage of disease	
IIIB IV	28 (30.8%) 63 (69.2%)
Performance status	
PS = 0 PS ≥ 1	12 (13.2%) 79 (86.8%)
Weight loss before CTH	
Yes No No data	39 (42.9%) 43 (47.2%) 9 (9.9%)
Anemia before CTH	
Yes No	59 (64.8%) 32 (35.2%)
Number of cycles of I line CTH	
Median Mean ± std. dev.	4 3.4 ± 0.9
Radiotherapy	
Yes No	19 (20.9%) 72 (79.1%)
Subsequent lines of treatment	
Yes No	51 (56%) 40 (44%)
Second-line chemotherapy (monotherapy)	51 (56.1%)
ERL PEM DCX	12 (13.2%) 26 (28.6%) 13 (14.3%)
Third-line chemotherapy (monotherapy)	15 (16.5%)
ERL PEM DCX	5 (5.5%) 6 (6.6%) 4 (4.4%)

### Methods

**Fig. 1:**
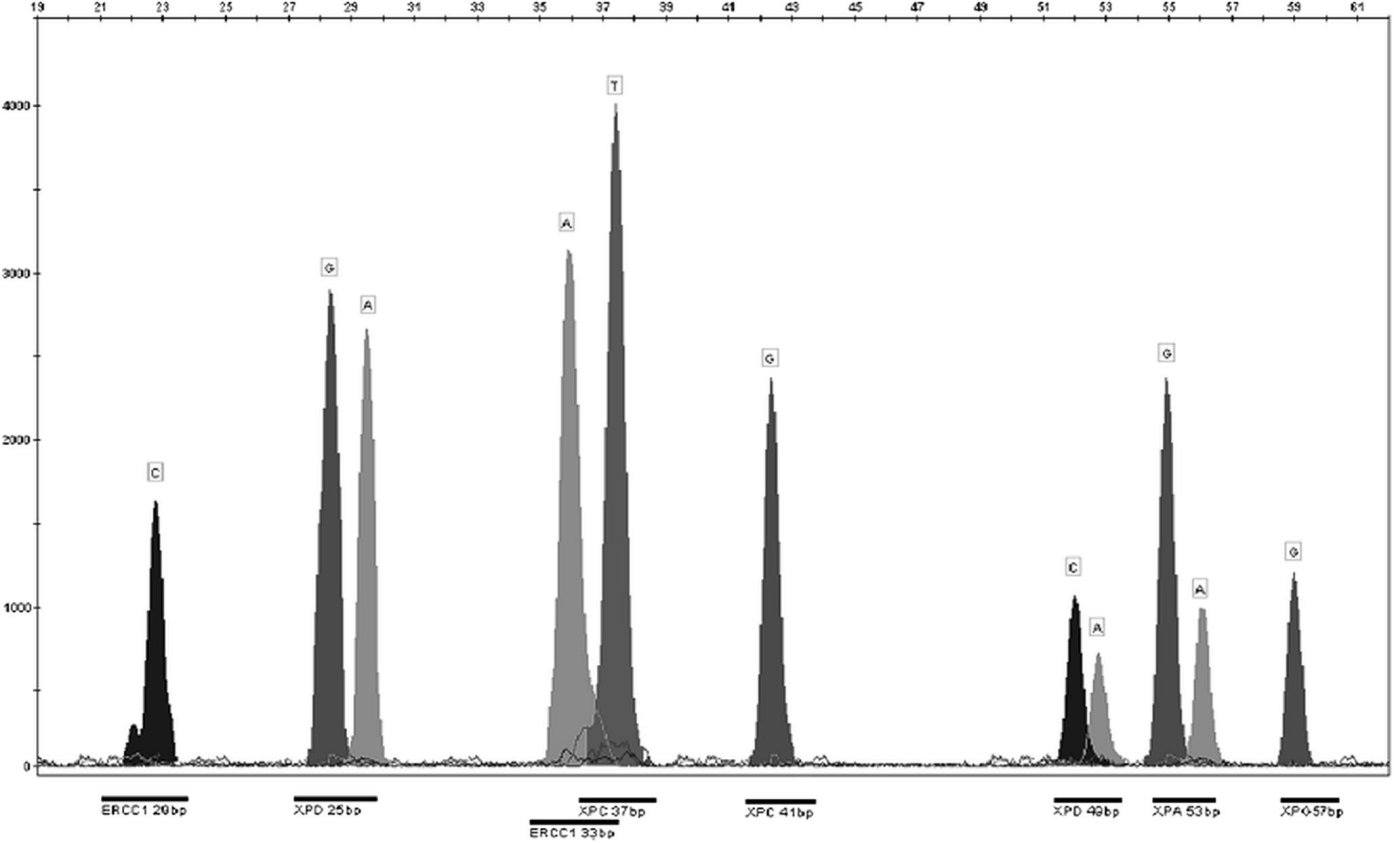
Genotyping results (capillary electrophoresis of SNaPshot® PCR products) From left: CC homozygote (8092C > A), AG heterozygote (934G > A), AA (19007C > T), TT (1385C > T) and GG (2704C > A) homozygotes, AC heterozygote (2251A > C), AG heterozygote (−4A > G), GG homozygote (3310C > G) (in certain cases analysis performed on the opposite strand where A = T and G = C)

Approximately 5 ml of peripheral blood was collected from each patient. After isolation of DNA (QIAamp DNA Blood Mini Kit, Qiagen, Canada) using a spectrophotometer (BioPhotometer plus cuvette equipped with UV / VIS filters, Eppendorf, Germany), purity and quantity of the isolated nucleic acids were evaluated. The next step was the use of mini-sequencing technique for genotyping (ABI PRISM® SnaPshot® Multiplex Kit, Life Technologies). An example of the genotyping result is shown in Fig. 1.

Response to therapy was evaluated according to the RECIST (Response Evaluation Criteria in Solid Tumors) criteria (v.1.1). Common Toxicity Criteria (CTC) guideline (version no. 4.03) was used for toxicity assessment. Response to therapy, progression-free survival (PFS) and overall survival (OS) were correlated with demographic, clinical and genetic factors.

### Statistical Analysis

Statistical analysis was performed using MedCalc 10 (MedCalc Software, Belgium). Results of *p* < 0.05 were considered as statistically significant. Hardy-Weinberg Equilibrium Eq. (HW) and the correlation between selected clinical and demographic factors and SNPs were assessed by Chi Square (χ^2^) test. Using the Kaplan-Meier method, the probability of PFS and OS depending on clinical factors, demographic factors and SNP variants was evaluated. The factors potentially affecting survival were evaluated using Cox regression model with a stepwise selection and minimum AIC factor (Akaike Information Criterion).

## Results

The distribution of demographic and clinical characteristics (age, gender, smoking status, histology, PS and stage of disease) was independent of SNPs variants. The characteristics of the studied SNPs was shown in Table 2. The genotypes of all the examined genes were in Hardy-Weinberg equilibrium, except for the 2251A > C SNP of *XPD/ERCC2* gene (*p* = 0.0019, χ^2^ = 9.679).

**Table 2:** Characteristics of the studied single nucleotide polymorphisms

No.	Gene	Localisation: chromosome; exon	DNA change	mRNA/5’UTR* change	HGSV	Amino acid change		MAF	rs number
1.	*ERCC1*	Chr 19; Ex. 8/9	G > A	19007C > T	g.45923653 A > G	Asn118Asn	N > N	A = 36.3%	rs11615
2.	*ERCC1*	Chr 19; Ex. 1/9	G > T	8092C > A	g.45912736 C > A	Gln504Lys	Q > K	A = 29.2%	rs3212986
3.	*XPD/ERCC2*	Chr 19; Ex. 1/23	T > G	2251A > C	g.45854919 T > G	Lys751Gln	K > Q	G = 23.7%	rs13181
4.	*XPD/ERCC2*	Chr 19; Ex. 15/23	C > T	934G > A	g.45867259 C > T	Asp312Asn	D > N	*T* = 19.4%	rs1799793
5.	*XPA*	Chr 9; 5’UTR	C > T	-4A > G*	g.100459578 T > C	–	–	*T* = 34.7%	rs1800975
6.	*XPC*	Chr 3; Ex. 8/16	G > A	1385C > T	g.14199887 G > A	Val499Ala	A > V	A = 24.8%	rs2228000
7.	*XPC*	Chr 3; Ex. 16/16	T > G	2704C > A	g.14187449 G > T	Lys939Glu	Q > K	G = 34.4%	rs2228001
8.	*XPG/ERCC5*	Chr 3; Ex. 23/23	G > C	3310C > G	g.103528002G > C	Asp1558His	D > H	C = 37.7%	rs17655

*HGSV* Human Genome Variation Society, *MAF* minor allele frequency

### Response to Treatment

In the study population, there was no case of complete remission (CR). Progression of the disease (PD) was recorded in 45.1% of patients, whereas stable disease (SD) and partial response (PR) were observed in 17.6 and 37.3% of patients, respectively (therefore, control of the disease was achieved in 54.9%). For patients with worse performance status (PS), a higher risk of disease progression was reported (PS ≥ 1; OR = 4.9, 95%CI: 1.00–23.69; *p* = 0.0495). Also, when squamous cell carcinoma (OR = 3.71, 95%CI: 1.07–12.89; *p* = 0.0392) or the presence of anemia before starting chemotherapy (OR = 3.03, 95%CI: 1.20–7.64; *p* = 0.0189) was diagnosed a significantly higher risk of early progression was observed. As regards other demographic and clinical factors, there was no statistically significant difference in the response to treatment when first-line chemotherapy was used (Table 3). Moreover, in the case of all studied SNPs, non-significant effect on response to treatment was recorded (Table 4).

**Table 3:** Influence of demographic and clinical factors on: response rates, progression-free survival and overall survival in the study group

Variable	PD 41 (45.1%)	SD, PR 50 (54.9%)	*p*, OR 95%CI	Median PFS (mo.)	*p,* χ^2^	HR 95%CI	Median OS (mo.)	*p,* χ^2^	HR (95%CI)
Sex									
Male Female	31 (50.8%) 10 (33.3%)	30 (49.2%) 20 (66.7%)	0.1179, 0.4839 0.1948–1.2022	4 6	0.8653 0.0288	0.9560 0.5684–1.6079	13 11	0.7280 0.1210	1.1177 0.5971–2.0923
Age (years)									
≤ 70 > 70	34 (46.6%) 7 (38.9%)	39 (53.4%) 11 (61.1%)	0.5581, 0.7299 0.2546–2.0929	3.5 6	0.6344 0.2262	0.8719 0.4956–1.5339	11.5 16.5	0.7812 0.0772	0.9061 0.4519–1.8169
Smoking status									
Smokers Non-smokers	39 (45.9%) 2 (40%)	46 (54.1%) 3 (60%)	0.7978, 0.7863 0.125–4.9481	4 7.5	0.5347 0.3854	0.7441 0.2926–1.8921	11.5 18.5	0.8851 0.0209	0.9187 0.2909–2.9012
Histopathological diagnosis									
Adenocarcinoma	18 (40%)	27 (60%)	–	6	0.1356	–	12	0.6073	–
Squamous cell carcinoma	10 (71.4%)	4 (28.6%)		2	5.5517		6.5	1.8352	
Largecel carcinoma	8 (47.1%)	9 (52.9%)		4			13		
NOS NSCLC	5 (33.3%)	10 (66.7%)		3.5			9.5		
Adenocarcinoma Other	18 (39.1%) 23 (51.1%)	28 (60.9%) 22 (48.9%)	0.2520, 1.6263 0.7077–3.7371	6 3	*0.0456* *3.9962*	1.6464 1.010–2.6846	12 11.5	0.4094 0.6805	1.2817 0.7107–2.3116
Non-squamous cell carcinoma Squamous cell carcinoma	31 (40.3%) 10 (71.4%)	46 (59.7%) 4 (28.6%)	*0.0392, 3.7092* *1.0673–12.8866*	4.5 2	*0.0140* *6.0383*	2.9420 1.2441–6.9589	12 6.5	0.1807 1.7916	1.8864 0.7448–4.7778
Large cell carcinoma Other	8 (50%) 33 (%)	8 (50%) 42 (%)	0.6619, 0.7857 0.2666–2.3157	3.75 4	0.4972 0.4608	0.8030 0.4263–1.5128	13 11.5	0.9921 0.0001	1.0036 0.4928–2.0439
Stage of disease									
IIIB IV	10 (35.7%) 31 (49.2%)	18 (64.3%) 32 (50.8%)	0.2348, 1.7438 0.6968–4.3641	7 3	*0.0094* *6.7519*	1.9186 1.1737–2.3458	18 10	0.0701 3.2800	1.7584 0.9546–3.2383
Performance status									
PS = 0 PS ≥ 1	2 (16.7%) 39 (49.4%)	10 (83.3%) 40 (50.6%)	*0.0495, 4.8750* *1.0031–23.691*	7.5 3.5	0.2694. 1.2197	1.4237 0.7606–2.6652	21 11	0.0700 3.2831	1.9516 0.9468–4.0241
Weight loss before chemotheapy									
No Yes	19 (44.2%) 17 (43.6%)	24 (55.8%) 22 (56.4%)	0.9567, 0.9761 0.4075–2.3375	6 3	0.7040 0.1443	1.1040 0.6626–1.8396	18 7.5	*0.0376* *4.3237*	2.1589 1.0451–4.4603
Anemia before chemotherapy									
No Yes	9 (28.1%) 32 (54.2%)	23 (71.9%) 27 (%45.8)	*0.0189, 3.0284* *1.2006–7.6394*	6.5 3	*0.0154* *5.8656*	1.8416 1.1234–3.0184	13 11	0.3844 0.7565	1.3012 0.7189–2.3551
Radiotherapy									
Yes No	7 (36.8%) 34 (47.2%)	12 (63.2%) 38 (52.8%)	0.4204, 1.5338 0.5418–4.3425	3 4.5	0.4613 0.5426	0.8126 0.4679–1.4114	18 11	0.0834 2.9982	0.5429 0.2719–1.0839
Subsequent lines of treatment									
Yes No	–	–	–	5 3	0.7157 0.1326	1.0952 0.6713–1.7870	16.5 8	*0.0305* *4.6802*	1.9956 1.0671–3.7313

Statistically significant results were marked by italics

**Table 4:** Influence of studied single nucleotide polymorphisms on: response rates, progression-free survival and overall survival in the study group

Variable	PD 41 (45.1%)	SD, PR 50 (54.9%)	*p*, OR 95%CI	Median PFS (mo.)	*p,* χ^2^	HR 95%CI	Median OS (mo.)	*p,* χ^2^	HR (95%CI)
*ERCC1* (19007C > T)									
CC CT TT	7 (63.6%) 22 (47.8%) 12 (35.3%)	4 (36.4%) 24 (52.2%) 22 (64.7%)	–	2.5 5 4.5	0.2440 2.8212	–	8 11.5 13	0.3551 2.0708	–
CC CT lub CT	7 (63.6%) 34 (42.5%)	4 (36.4%) 46 (57.5%)	0.1958, 0.4224 0.1144–1.5591	4.5 2.5	0.0781 3.1037	2.1093 (0.9194–4.8389)	12 8	0.8144 0.0551	1.1253 (0.4199–3.0157)
CT CC lub TT	22 (47.8%) 19 (42.2%)	24 (52.2%) 26 (57.8%)	0.5914, 0.7972 0.3485–1.8235	3.5 5	0.4187 0.6540	0.8191 (0.5050–1.3285)	13 11.5	0.1939 1.6876	1.4714 (0.8216–2.6352)
TT CC lub CT	12 (35.3%) 29 (50.9%)	22 (64.7%) 28 (49.1%)	0.1506, 1.8988 0.7921–4.5519	3.5 4.5	0.8228 0.0502	0.9447 (0.5739–1.5548)	11.5 13	0.1429 2.1464	0.6416 (0.3543–1.1618)
*ERCC1* (8092C > A)									
AA AC CC	0 (0%) 14 (41.2%) 27 (48.2%)	1 (100%) 20 (58.8%) 29 (51.8%)	–	3.5 5.5 3	0.5597 1.1608	–	5.5 13 10	0.2313 2.9278	–
AA AC lub CC	- 41 (45.6%)	1 (100%) 49 (54.4%)	0.5754, 2.5152 0.0998–63.4007	3.5 4	0.6188 0.2476	1.9386 (0.1429–26.302)	5.5 12	0.1352 2.2313	20.103 (0.3919–1031)
AC AA lub CC	14 (41.2%) 27 (47.4%)	20 (58.8%) 30 (52.6%)	0.5661, 1.2857 0.5449–3.0334	5.5 3.5	0.2887 1.1259	0.7713 (0.4774–1.2461)	13 10	0.3295 0.9507	0.7456 (0.4133–1.3451)
CC AA lub AC	27 (48.2%) 14 (40%)	29 (51.8%) 21 (60%)	0.4442, 0.7160 0.3043–1.6847	3 5	0.3337 0.9345	1.2660 (0.7848–2.0424)	10 13	0.4535 0.5619	1.2526 (0.6952–2.2569)
*XPD/ERCC2* (2251A > C)									
AA AC CC	16 (47.1%) 17 (37%) 6 (85.7%)	18 (52.9%) 29 (63%) 1 (14.3%)	–	3 6 2	0.0834 4.9682	–	9.5 13 7.5	0.3808 1.9307	–
AA AC lub CC	16 (47.1%) 23(43.3%)	18 (52.9%) 30 (56.7%)	0.7376, 0.8625 0.3631–2.0489	3 4.5	0.3878 0.7458	1.2508 (0.7527–2.0783)	9.5 12	0.6023 0.2716	1.1822 (0.6299–2.2187)
AC AA lub CC	17 (37%) 22 (53.6%)	29 (63%) 19 (46.4%)	0.1197, 1.9752 0.838–4.656	6 2.5	0.0843 2.9793	0.6458 (0.3931–1.0611)	13 9	0.2919 1.1108	0.7184 (0.3884–1.3289)
CC AA lub AC	6 (85.7%) 33 (41.2%)	1 (14.3%) 47 (58.8%)	0.0519, 0.1170 0.0135–1.0181	2 4.5	*0.0444* *4.0430*	3.1953 (1.0297–9.9150)	7.5 12	0.2268 1.4610	2.3198 (0.5927–9.0790)
*XPD/ERCC2* (934G > A)									
AA AG GG	3 (50%) 23 (43.4%) 13 (43.3%)	3 (50%) 30 (56.6%) 17 (56.7%)	–	3 4 5.5	0.9578 0.0862	–	7.5 13 9.5	0.7312 0.6263	–
AA AG lub GG	3 (50%) 36 (43.7%)	3 (50%) 47 (56.3%)	0.7526, 0.7660 0.1459–4.021	3 4.5	0.8002 0.0641	0.8878 (0.3533–2.2310)	7.5 12	0.5884 0.2929	1.4599 (0.3709–5.7466)
AG AA lub GG	23 (43.4%) 16 (44.4%)	30 (56.6%) 20 (55.6%)	0.9221, 1.0435 0.4448–2.4482	4 5.5	0.7969 0.0662	1.0665 (0.6530–1.7420)	13 9.5	0.6728 0.1783	1.1377 (0.6251–2.0705)
GG AA lub AG	13 (43.3%) 26 (44%)	17 (56.7%) 33 (56%)	0.9474, 1.0303 0.4246–2.4998	5.5 4	0.8983 0.0163	0.9672 (0.5800–1.6130)	9.5 13	0.4971 0.4612	0.8075 (0.4356–1.4969)
*XPC* (1385C > T)									
CC CT TT	27 (45%) 11 (47.8%) 3 (42.9%)	33 (55%) 12 (52.2%) 4 (57.1%)	–	4 4 5.5	0.8494 0.3264	–	13 10 14.75	0.8497 0.3256	–
CC CT lub TT	27 (45%) 14 (46.6%)	33 (55%) 16 (53.4%)	0.8810, 1.0694 0.4439–2.5766	4 5	0.4134 0.6690	1.2325 (0.7469–2.0339)	10 13.5	0.8706 0.0265	1.0515 (0.5748–1.9233)
CT CC lub TT	11 (47.8%) 30 (44.7%)	12 (52.2%) 37 (55.3%)	0.8000, 0.8845 0.3423–2.2856	4 4.5	0.5456 0.3653	1.1893 (0.6778–2.0869)	10 13	0.5673 0.3273	1.2141 (0.6246–2.3596)
TT CC lub CT	3 (42.9%) 38 (45.7%)	4 (57.1%) 45 (54.3%)	0.8814, 1.1259 0.2371–5.3474	5.5 4	0.9490 0.0041	0.9698 (0.3788–2.4830)	14.75 11	0.8323 0.0448	0.8978 (0.3309–2.4355)
*XPC* (2704C > A)									
AA AC CC	5 (62.5%) 14 (45.2%) 15 (39.5%)	3 (37.5%) 17 (54.8%) 23 (60.5%)	–	3 5.5 3.5	0.2529 2.7495	–	10 18 10	0.1663 3.5882	–
AA AC lub CC	5 (62.5%) 29 (49.1%)	3 (37.5%) 30 (50.9%)	0.4823, 0.5800 0.1269–2.6510	3 4	0.1684 1.8970	1.9578 (0.7526–5.0927)	10 13	0.2498 1.3245	1.8701 (0.6440–5.4306)
AC AA lub CC	14 (45.2%) 20 (43.4%)	17 (54.8%) 26 (56.5%)	0.8840, 0.9341 0.3735–2.336	5.5 3.5	0.1487 2.0856	0.6762 (0.3976–1.1500)	18 10	0.0669 3.3570	0.5442 (0.2839–1.0433)
CC AA lub AC	15 (39.5%) 19 (48.7%)	23 (60.5%) 20 (51.3%)	0.4148, 1.4567 0.5898–3.5976	3.5 4	0.5036 0.4474	1.1954 (0.7086–2.0166)	10 16.5	0.2569 1.2854	1.4591 (0.7593–2.8037)
*XPA* (−4A > G)									
AA AG GG	4 (44.4%) 23 (48.9%) 13 (40.6%)	5 (55.6%) 24 (51.1%) 19 (59.4%)	–	6 3.5 5.5	0.9913 0.0174	–	18 11 13	0.4931 1.4142	–
AA AG lub GG	4 (44.4%) 36 (45.5%)	5 (55.6%) 43 (54.5%)	0.9488, 1.0465 0.2613–4.1905	6 4	0.9047 0.0143	1.0494 (0.4766–2.3105)	18 11.5	0.2752 1.1905	0.6012 (0.2410–1.4996)
AG AA lub GG	23 (48.9%) 17 (41.4%)	24 (51.1%) 24 (58.6%)	0.4829, 0.7391 0.3177–1.7198	3.5 5.5	0.9842 0.0004	1.0050 (0.6137–1.6458)	11 18	0.3575 0.8467	1.3213 (0.7299–2.3919)
GG AA lub AG	13 (40.6%) 27 (48.2%)	19 (59.4%) 29 (51.8%)	0.4921, 1.3607 0.565–3.277	5.5 3.5	0.9209 0.0098	0.9741 (0.5808–1.6338)	13 11.5	0.8210 0.0512	0.9294 (0.4929–1.7523)
*XPG/ERCC5* (3310C > G)									
CC GC GG	29 (46%) 11 (42.3%) -	34 (54%) 15 (57.7%) 1 (100%)	–	4 4 4.5	0.7988 0.4493	–	11.5 13 -	0.7334 0.6202	–
CC CG lub GG	29 (46%) 11 (40.7%)	34 (54%) 16 (59.3%)	0.6437, 0.8060 0.3233–2.0098	4 4	0.5142 0.4255	0.8389 (0.4949–1.4221)	11.5 13	0.7580 0.0949	0.9007 (0.4631–1.7518)
CG CC lub GG	11 (42.3%) 29 (45.3%)	15 (57.7%) 35 (54.7%)	0.7949, 1.1299 0.45–2.837	4 4.5	0.5628 0.3349	1.1704 (0.6869–1.9942)	13 11.5	0.6435 0.2142	1.1722 (0.5982–2.2971)
GG CC lub CG	- 40 (44.9%)	1 (100%) 49 (55.1%)	0.5855, 2.4545 0.0973–61.8949	4.5 4	0.7135 0.1348	1.5818 (0.1368–18.2947)	- 11.5	0.4985 0.4581	0.3536 (0.0174–7.1790)

Statistically significant results were marked by italics

### Progression-Free Survival

Median PFS for the study population was 4 months. Clinical factors associated with a shortened PFS in the study group were as follows: poor PS (2 vs 6 months; HR = 3.03, 95%CI: 1.61–5.88; *p* = 0.0006); anemia before chemotherapy (3 vs 6.5 months.; HR = 1.84, 95%CI: 1.12–3.02; *p* = 0.0154); stage IV of the disease (3 vs 7 months; HR = 1.92, 95%CI: 1.18–3.12; *p* = 0.0094), diagnosis of non-adenocarcinoma (3 vs 6 months; HR = 1.64, 95%CI: 1.01–2.68; p = 0,0456) or squamous cell carcinoma (2 vs 4.5 months; HR = 2.94, 95%CI: 1.24–6.96; *p* = 0.0140). Other evaluated clinical factors had no significant influence on PFS.

**Fig. 2:**
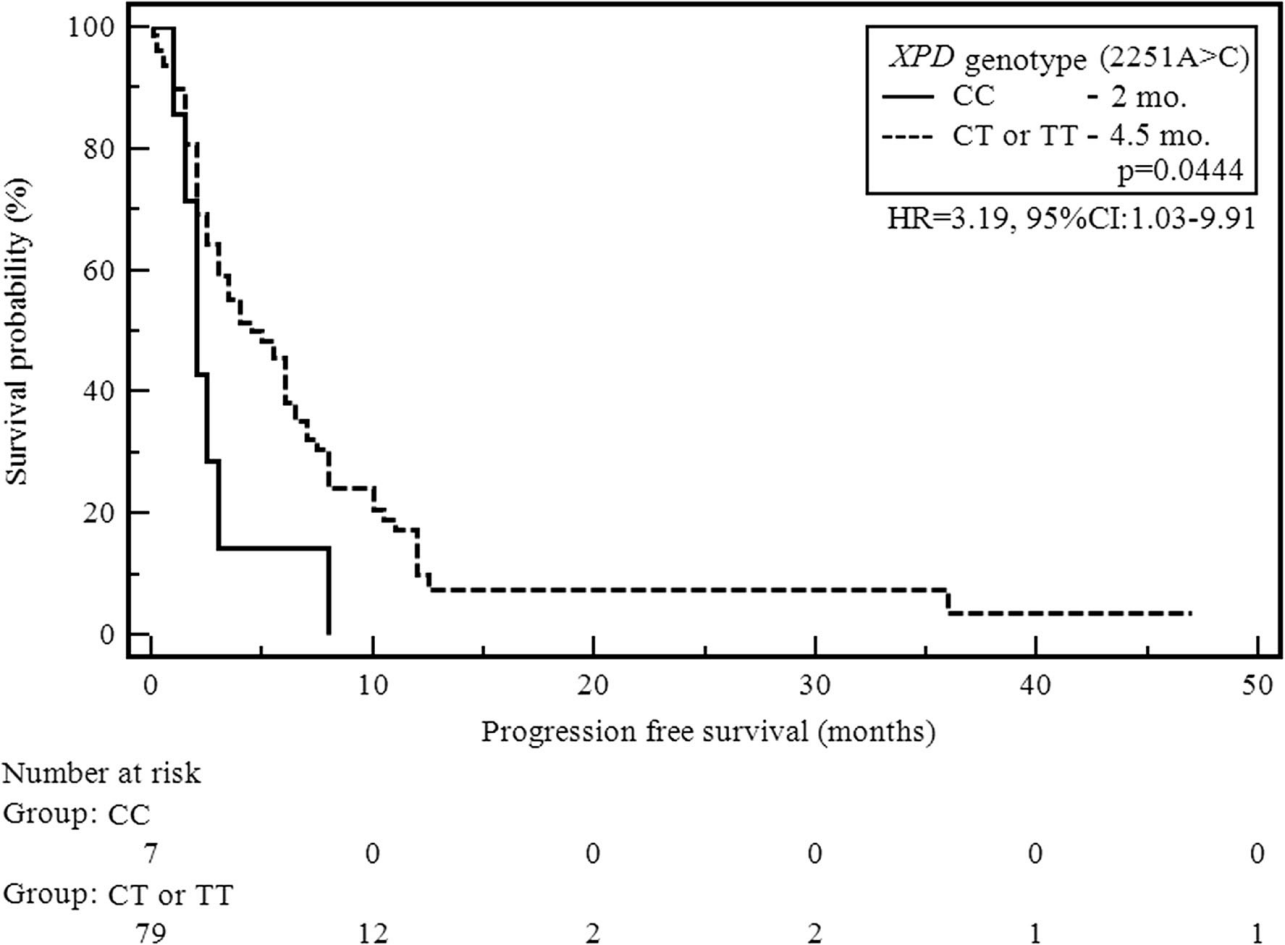
The probability of progression-free survival alteration depending on *XPD/ERCC2* (2251A > C) genotype

In patients with the CC genotype (2251A > C) of the *XPD/ERCC2* gene, a significant decrease in median PFS, compared to patients with other polymorphic variants of this gene, was observed (2 vs 4.5 months; HR = 3.19, 95% CI: 1.03–9.91; *p* = 0.0444; Fig. 2). Statistically significant correlation between the duration of the PFS and the occurrence of individual genotypes was not demonstrated for the remaining studied SNPs. Detailed results regarding the impact of the demographic, clinical and genetic factors on PFS were shown in Tables 3 and 4.

With the use of Cox multivariate logistic regression, we have demonstrated that factors which significantly shortened PFS in patients treated with cisplatin/gemcitabine based chemotherapy (overall fit of the model; χ^2^ = 46.59; *p* = 0.0025) were the following: poor PS (PS = 2, HR = 5.78, 95%CI: 2.18–15.34; *p* = 0.0005), higher stage of progression (IV, HR = 3.21, 95%CI: 1.41–7.28; *p* = 0.0055), diagnosis of non-adenocarcinoma type of NSCLC (HR = 3.14, 95%CI: 1.14–8.61; *p* = 0.0270) and CC genotype (2251A > C) of *XPD/ERCC2* gene (HR = 12.62, 95%CI: 1.23–129.43; *p* = 0.0337).

### Overall Survival

**Fig. 3:**
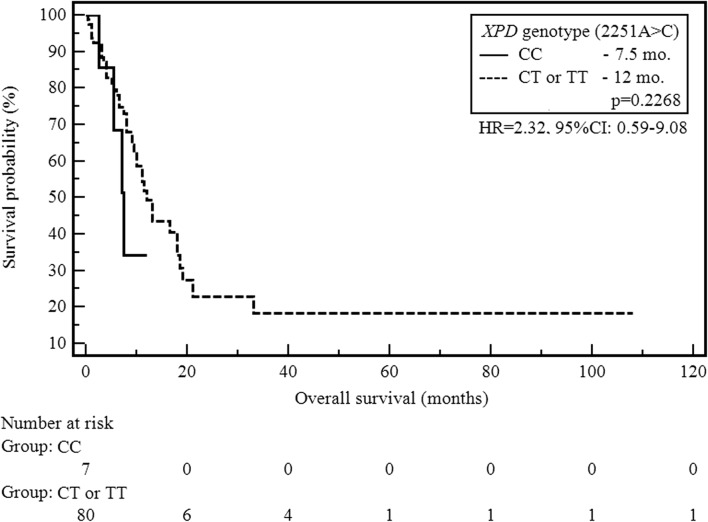
The probability of overall survival alteration depending on *XPD/ERCC2* (2251A > C) genotype

Median OS for the study population was 12 months. Clinical factors associated with a shortened OS in the study group were: weight loss before the beginning of chemotherapy (7.5 vs 18 months; HR = 2.16, 95%CI: 1.04–4.46; *p* = 0.0376) and lack of subsequent lines of treatment (8 vs 16.5 months; HR = 2, 95%CI: 1.07–3.73; *p* = 0.0305). Poor PS shows a trend of only borderline significance (11 vs 21 months; HR = 1.95, 95%CI: 0.95–4.02; *p* = 0.07). Univariate analysis demonstrated that there was no significant correlation with the other studied clinical factors as well as SNPs and the length of OS. The influence of the polymorphism of *XPD/ERCC2* on OS was shown in Fig. 3.

With the use of Cox multivariate logistic regression, we have demonstrated that factors which significantly shortened OS in patients treated with cisplatin/gemcitabine based chemotherapy (overall fit of the model; χ^2^ = 49.98; *p* = 0.0006) were the following: poor PS (PS = 2, HR = 7.31, 95%CI: 2.17–24.61; *p* = 0.0014), higher stage of progression (IV, HR = 3.96, 95%CI: 1.15–13.55; *p* = 0.0294), diagnosis of squamous cell type of NSCLC (HR = 6.79, 95%CI: 1.80–25.60; *p* = 0.0049), age below 70 years (HR = 7.39, 95%CI: 1.36–40,25; *p* = 0.0214), anemia before the beginning of chemotherapy (HR = 6.07, 95%CI: 1.36–27.09, *p* = 0.0188), lack of subsequent lines of chemotherapy (HR = 18.46, 95%CI: 4.31–78.95; *p* < 0.0001) and CC genotype (2251A > C) of *XPD/ERCC2* gene (HR = 51.99, 95%CI: 1.19–2274.11; *p* = 0.0414).

Detailed data of response to treatment, PFS and OS were shown in Tables 3 and 4.

## Discussion

The role of DNA repair proteins (especially those participating in NER mechanism) in the removal of the cisplatin adducts and gemcitabine induced alterations (although to a lesser extent) is well known [7–9]. Occurrence of SNPs in non-coding (3’UTR, promoter regions) or coding sequences of genes may lead to numerous alterations including changes in the structure, stability, folding, expression and function of proteins [13]. Therefore, testing of SNPs has a high potential to be applied in routine clinical practice. Thus, a number of studies (unfortunately mainly retrospective) were aimed at assessing the correlation between various SNPs of genes involved in DNA repair and the effectiveness of different treatment regimens in patients with advanced NSCLC [10–12, 14, 15]. In the available literature. The most frequently evaluated SNPs of *XPD/ERCC2* gene, which can potentially be related to the effectiveness of chemotherapy based on platinum compounds and gemcitabine, were: 2251A > C and 934G > A [16–20]. A meta-analysis performed by Qin et al. on the basis of 24 studies (4468 patients with NSCLC) assessed the impact of both of the above described SNPs on treatment response, PFS, and OS in patients treated with first-line chemotherapy based on platinum compounds and next generation drug [21]. Sixteen studies considered objective response to first-line chemotherapy. They demonstrated that the SNP 2251A > C was not significantly correlated with the objective response to treatment. However, the results of an additional subgroup analysis showed a significant difference in response to treatment depending on the patients’ race. In the case of Asians (8 studies, 1795 patients) no significant association between genotype variant and the possibility of obtaining the response to treatment was noted, while in Caucasians (8 studies, 853 patients) beneficial effect of AA genotype was observed (OR = 1.35, 95% CI: 1–1,83, *p* = 0.05) [21]. Authors did not find significant differences in the length of PFS in carriers of various genotypes of *XPD/ERCC2* gene (SNP 2251A > C). Subgroup analysis showed a significant increase in the risk of early progression in C allele carriers (CC or CA) compared to patients with AA genotype in Asian patients (HR = 1.39, 95% CI: 1.07–1.81, *p* = 0.015). However, no such correlation was found in Caucasians [21]. Also no significant differences in the length of OS depending on the occurrence of SNP 2251A > C of *XPD/ERCC2* gene were recorded. Similarly, subgroup analysis showed no significant increase in the risk of early death in carriers of C allele compared to patients with the AA genotype in both Asians and Caucasians [21]. In the above meta-analysis, patients were treated with different regimens. This undoubtedly had an impact on the obtained results and can lead to deceptive conclusions. In addition, this makes it difficult to directly compare our results (only 3 studies concerned Caucasian patients treated exclusively with cisplatin / gemcitabine regimen). Interestingly, in this study, we demonstrated that in the case of response to treatment and PFS, our results are consistent with those obtained in Asian population and not in Caucasians. However, in the case of OS, our data are in conformity with literature regardless of the race of the patients [21]. Although we have taken efforts to design and perform research in comprehensive and accurate manner, our study has some limitations: population of patients enrolled in the study is heterogeneous; selected patients received not only first-line chemotherapy but also radiotherapy, whereas selected patients received subsequent lines of treatment.

In this study, we demonstrated a significantly higher risk of shortening the duration of PFS in carriers of CC genotype (2251A > C) of *XPD/ERCC2* gene treated with first-line chemotherapy based on combination of cisplatin and gemcitabine. Interestingly, this is consistent with the available literature data for Asian, but not Caucasian (same as ours) patients [21].

Therefore, we believe that the conclusive answer to the question whether the SNPs of *XPD/ERCC2* gene can be a useful predictor of chemotherapy based on a combination of platinum compounds and gemcitabine remains unknown. This issue requires further research on sufficiently large and homogeneous groups of patients. First of all, they should be treated with only one therapy regimen.

## Conclusion

Rare CC genotype of *XPD/ERCC2* gene may be considered as an unfavorable predictive factor for cisplatin/gemcitabine based chemotherapy in patients with advanced NSCLC.
